# FluoSpec 2—An Automated Field Spectroscopy System to Monitor Canopy Solar-Induced Fluorescence

**DOI:** 10.3390/s18072063

**Published:** 2018-06-28

**Authors:** Xi Yang, Hanyu Shi, Atticus Stovall, Kaiyu Guan, Guofang Miao, Yongguang Zhang, Yao Zhang, Xiangming Xiao, Youngryel Ryu, Jung-Eun Lee

**Affiliations:** 1 Department of Environmental Sciences, University of Virginia, Charlottesville, VA 22904, USA; hs9hj@virginia.edu (H.S.); aes2aj@virginia.edu (A.S.); 2 Department of Natural Resources and Environmental Sciences, University of Illinois at Urbana and Champaign, Champaign, IL 61801, USA; kaiyug@illinois.edu (K.G.); guofang.miao@gmail.com (G.M.); 3 National Center of Supercomputing Applications, University of Illinois at Urbana and Champaign, Champaign, IL 61801, USA; 4 Jiangsu Provincial Key Laboratory of Geographic Information Science and Technology, International Institute for Earth System Sciences, Nanjing University, Nanjing 210023, China; yongguang_zhang@nju.edu.cn; 5 Jiangsu Center for Collaborative Innovation in Geographical Information Resource Development and Application, Nanjing 210023, China; 6 Department of Microbiology and Plant Biology, Center for Spatial Analysis, University of Oklahoma, Norman, OK 73019, USA; zy2309@columbia.edu (Y.Z.); xiangming.xiao@ou.edu (X.X.); 7 Department of Landscape Architecture and Rural Systems Engineering, Seoul National University, Seoul 08826, Korea; ryuyr77@gmail.com; 8 Department of Earth, Environment, and Planetary Sciences, Brown University, Providence, RI 02912, USA; leeje@brown.edu

**Keywords:** photosynthesis, proximal remote sensing, spectroscopy, vegetation

## Abstract

Accurate estimation of terrestrial photosynthesis has broad scientific and societal impacts. Measurements of photosynthesis can be used to assess plant health, quantify crop yield, and determine the largest CO_2_ flux in the carbon cycle. Long-term and continuous monitoring of vegetation optical properties can provide valuable information about plant physiology. Recent developments of the remote sensing of solar-induced chlorophyll fluorescence (SIF) and vegetation spectroscopy have shown promising results in using this information to quantify plant photosynthetic activities and stresses at the ecosystem scale. However, there are few automated systems that allow for unattended observations over months to years. Here we present FluoSpec 2, an automated system for collecting irradiance and canopy radiance that has been deployed in various ecosystems in the past years. The instrument design, calibration, and tests are recorded in detail. We discuss the future directions of this field spectroscopy system. A network of SIF sensors, FluoNet, is established to measure the diurnal and seasonal variations of SIF in several ecosystems. Automated systems such as FluoSpec 2 can provide unique information on ecosystem functioning and provide important support to the satellite remote sensing of canopy photosynthesis.

## 1. Introduction

Terrestrial vegetation photosynthesis contributes to the largest CO_2_ flux on Earth [1]. Remote monitoring of vegetation photosynthesis can provide information on the spatial and temporal variability of photosynthesis and its biotic and abiotic controls [2,3]. The widely-used eddy covariance (EC) technique provides a measure of the net ecosystem exchange (NEE) which can be used to estimate the ecosystem level photosynthesis: the Gross Primary Productivity (GPP) [4]. Yet, calculation of GPP based on the EC heavily relies on the assumption of the respiration–temperature relationship, which may overestimate the daytime respiration, and could potentially lead to an overestimation of GPP [5]. Independent and long-term observations of canopy photosynthesis using vegetation spectroscopy are thus needed. An additional benefit of this approach is its ability to link with satellite observations, and thus can be up-scaled to regional and global scales [6,7]. Recent developments in solar-induced chlorophyll fluorescence (SIF) suggest that it is an indicator of canopy photosynthesis [2,8,9,10,11,12,13,14]. Long-term and high frequency measurements of SIF that cover a wide variety of terrestrial ecosystems are needed for multiple reasons: (1) high temporal frequency measurements of canopy photosynthesis (every a few minutes) provide important insights into canopy responses to environmental variables and have the potential to reveal photo protection mechanisms [15,16]; (2) ground observations provide a network of sensors that can be used to validate satellite data [2,17,18] and also scale up photosynthesis to regional and global scales [19,20,21]; and (3) SIF observations can be used as additional constraints in addition to the EC fluxes to ecosystem models [22,23].

In recent decades, various types of proximal remote sensing instruments have been developed for monitoring different ecosystem functioning. In one of the first continuous measurements of SIF, Yang et al. [2] developed FluoSpec and made measurements over a temperate mixed forest. Cogliati et al. [24] developed a similar type of instrument that measures both SIF and canopy reflectance over an agricultural field and a grass field. Daumard et al. [25] measured SIF over a grain sorghum field and found that SIF and PRI (Photochemical Reflectance Index) are more sensitive to drought compared with NDVI. Pérez-Priego et al. [26], one of the earliest groups to measure canopy SIF, showed that plants under water stress have lower SIF. Adopting the Differential Optical Absorption Spectroscopy (DOAS) concepts, PhotoSpec has been developed recently, and it measures both the red and far-red fluorescence [27]. In addition to SIF systems, automated field instruments have been developed to measure multi-angle canopy reflectance [28,29]. AMSPEC (Automated, Multi-angle SPECtro-radiometer) has been deployed in several ecosystems and has provided insights in ecosystem light use efficiency [18,30,31]. Upward-looking cameras have been used to monitor the leaf area index (LAI) at an ecosystem scale in an oak-savanna ecosystem [32]. Light-emitting diode (LED) sensors have been developed as an economic and effective way to monitor canopy reflectance in an annual grassland [33]. These methods utilize the electromagnetic radiation in the visible and near-infrared region (0.4–1.1 μm). Continuous measurements of full canopy spectra (0.4–2.5 μm) are still rare, even though they provide additional information on leaf traits, like nitrogen and lignin concentration [34,35]. Thermal cameras or point infrared thermometers have been used to measure canopy temperature for a few decades, and continuous measurement of canopy temperature with a camera has been demonstrated recently [36]. These studies demonstrated both the importance and the technical difficulties of long-term observations. With growing interest from the scientific community [37], it is important to provide a detailed description of the SIF instrument that we are using at multiple sites.

These field SIF instruments are important tools for our understanding of the structural and environmental controls of photosynthesis and SIF. For example, Yang et al. [2] showed that the fluorescence yield is higher under cloudy days compared with sunny days. Migliavacca et al. [10] demonstrated that canopy structure is the dominant control of far-red fluorescence (specifically, SIF at 760 nm), while canopy biochemistry, such as the maximum carboxylation rate (Vcmax), and nitrogen concentration (%N) plays a secondary role. Liu et al. [38] analyzed the angular dependence of SIF emissions on a wheat field. Miao et al. [11] found that the SIF yield (SIF/APAR, where APAR is the absorbed photosynthetically active radiation) and light use efficiency (LUE = GPP/APAR) were negatively correlated over the course of a day. These measurements still only cover a small portion of the global biomes, and most of them are located in temperate ecosystems. To facilitate continuous and long-term observations, instruments that can be used by the entire scientific community are essential.

Networks of ground-based sensors have been proved to be essential to our understanding of how different ecosystems function and also provide a way of upscaling the results to the regional and global scales with the help of airborne or satellite remote sensing. For example, the FLUXNET is a network of EC towers that provide continuous measurements of land–atmosphere exchanges of CO_2_ and water [4]. The SpecNet is a network of optical sensors that collocate with EC towers, aiming to provide information on plant functioning [39]. A network of digital cameras has been established to monitor vegetation phenology, providing both observations of individual trees and a way to link ground observations to the satellite phenology data [40,41,42,43]. Individual sites of SIF measurements have been established in the past few years, but a network that synthesizes the efforts is still lacking.

Here, we describe the design, calibration, and field tests of the new version of FluoSpec, known as FluoSpec 2. FluoSpec 2 has a better temperature control, data collection flow, and signal-to-noise ratio. We document the instrument design, calibration, data acquisition, and lab and field tests. Using this instrument, we establish a network of SIF sensors (FluoNet). In the end, we discuss the future directions of field SIF system.

## 2. Materials and Methods

### 2.1. Instrument Design

**Figure 1:**
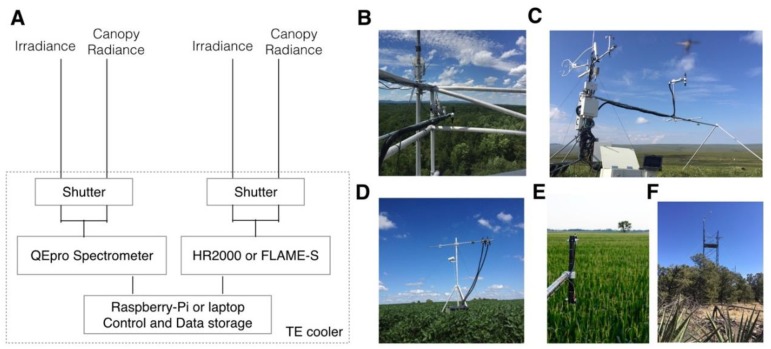
The design of FluoSpec 2. (**A**) An example of the system with both a solar-induced chlorophyll fluorescence (SIF) spectrometer and a reflectance spectrometer; (**B**) FluoSpec 2 on a 40-m tower in central Virginia; (**C**) Alaska site with FluoSpec 2; (**D**) FluoSpec 2 in a crop field in Illinois, and (**E**) Nebraska; (**F**) New Mexico.

The core element of FluoSpec 2 (Figure 1) is an ultra-high resolution spectrometer (QEpro, OceanOptics Inc., Dunedin, FL, USA) that is customized to measure radiation in the fluorescence emission wavelength range (650–800 nm). This represents the key difference between FluoSpec 2 and FluoSpec, which uses HR2000+, a spectrometer without temperature control and thus has a lower signal-to-noise ratio (~250:1). Given a specific grating (and thus the groove density), there is a trade-off between the spectral range and spectral resolution (Full-Width at Half Maximum, FWHM). Thus, in practice, to achieve a resolution that allows for SIF retrievals using narrow Fraunhofer lines, a spectral resolution close to ~0.1–0.3 nm is necessary [44]. A common misconception is to equate the spectral sampling interval with the spectral resolution. The spectral sampling interval is the difference in wavelength between two consecutive data points, which is usually half to one-third of the spectral resolution. Given a known spectral range (Δλ) and the number of pixels (N) of a spectrometer, we can calculate the dispersion (D’) of the spectrometer as:

D’ = Δλ/N,(1)

Since QEpro is not an imaging spectrometer, each pixel on the detector collect the photons from a specific wavelength. This is different from the nomenclature commonly used in the remote sensing community, in which pixels refer to a unit area on an image. We can calculate the spectral resolution (FWHM) as the product of dispersion and pixel resolution (P), which is a function of the slit size. Pixel resolutions of spectrometers are usually provided by the manufacturers. The slit size affects both FWHM (Equation (2)) and the signal received by the detector per second. A larger slit size means coarser FWHM but increases the number of photons that reach the detector per unit time and thus can reduce the integration time:

FWHM = D’ × P,(2)

In the meantime, key spectral windows where SIF is often retrieved need to be covered. In our case, we chose the near-infrared region (730–780 nm) where both the narrow Fraunhofer lines near 750 nm and the wide oxygen absorption band (O_2_–A, centered around 760 nm) are covered. The setting enables the retrieval of the NIR SIF at an FWHM of ~0.14 nm. If red SIF retrieval is needed, the spectrometer can be customized to cover the red region (650–730 nm). If the retrieval of both oxygen bands is intended, then the spectrometer can be customized to cover both O_2_A and O_2_B (centered around 687 nm) bands with a coarser resolution (~0.4 nm). An additional spectrometer (HR2000 or FLAME, OceanOptics Inc., Dunedin, FL, USA) can be added to the system to measure the canopy reflectance between 400 and 1100 nm at a spectral resolution of around 3 nm. The QEpro has an internal shutter which closes when dark current measurements are needed. As we will show later, routine dark signal measurements are not necessarily needed if the detector’s temperature and the integrating time are known.

QEpro has 1044 pixels, including 1024 active pixels, eight dummy pixels, and twelve optical dark pixels on the edge of the detector. The first four and last four pixels are intentionally blocked and thus, not optically active (i.e., do not respond to incoming radiation). Then, there are six pixels each after the first four and before the last four that are optically active but not used. We use only the 1024 pixels in the middle.

Measurements of both incoming solar radiation and canopy radiation are needed to retrieve SIF. FluoSpec 2 uses an optical shutter (FOS 2 × 2 TTL, OceanOptics Inc., Dunedin, FL, USA) and a fiber splitter to achieve this task. The spectrometer sends a programmed electronic signal (high or low) to the optical shutter, which opens one port at a time. Each port on the shutter is connected to one leg of the fiber splitter. On the other side of the shutter, ports are connected to fiber optics, one of which measures incoming radiation with a cosine corrector (CC-3, OceanOptics Inc., Dunedin, FL, USA) while the other one measures upwelling radiation from the vegetation canopy from a nadir view. The field-of-view of the downward-looking fiber is 25 degrees. The radius of the area that is measured by the system can then be calculated as h · tan(25/2), where h is the distance between the sensor and the vegetation canopy. When installing a sensor in an ecosystem with sparse canopies, we use a cosine corrector on the downward-looking fiber. In this way, a larger area can be observed, although it should be noted that leaves in the observed area do not contribute equally to the signal received by the sensor (weighted by the cosine of incident angle) [45].

An air conditioner is used to keep the enclosure at a stable temperature. Depending on the local weather and the desired cool down rate (degrees below ambient), the power need of the air conditioner varies. The power consumption of FluoSpec 2 without the air conditioner is ~15 W at its maximum (data not shown). Depending on the site location, the power need for the air conditioner varies and thus, the power consumption can vary from 50 W to 200 W. Adding a shading mechanism, installing the enclosure in the understory of a forest, or using a highly reflective surface will help to reduce the radiation load on the enclosure. QEpro has a TEC (Thermal Electric Cooler) that can further cool down the CCD (Charge-Coupled Device) detector to −10 °C. During the field measurements, we noticed that cooling down the system below zero Celsius when the relative humidity was high (>80%) caused condensation on the CCD detector. In addition to adding desiccant bags, three solutions were used: (1) before each round of measurement, we pre-heated the instrument to 30 Celsius, which caused the water droplets to evaporate. We then cooled down the instrument back to −10 Celsius for the measurements; (2) the TEC temperature was set to 0 Celsius to increase the dark current but eliminate the condensation. We found that the differences in the level of dark current were small—between −10 Celsius and 0 Celsius (see next section); and (3) an additional water tight enclosure was added to protect the QEpro spectrometer.

Several methods were adopted to increase the signal received by the spectrometer. First, the size of the fiber optics was increased from 600 μm to 1000 μm. Second, at the FOS 2 × 2 shutter, the focus of the fibers on each end of the port was adjusted to increase the light intensity. After these adjustments, the integration time for the bare fiber side facing vegetation to reach a digital number around 100,000 was around 0.8 s on a sunny day and 1.3~2 s for the cosine corrector that faces the sky. Cosine correctors, like other diffusers, cause significant signal loss and require a longer integration time. It should be noted that certain types of cosine corrector do not have a perfect cosine response, particularly when the incident angle from the sun is low, causing erroneous SIF retrievals when oxygen bands approaches are used (data not shown).

### 2.2. Data Acquisition

**Figure 2:**
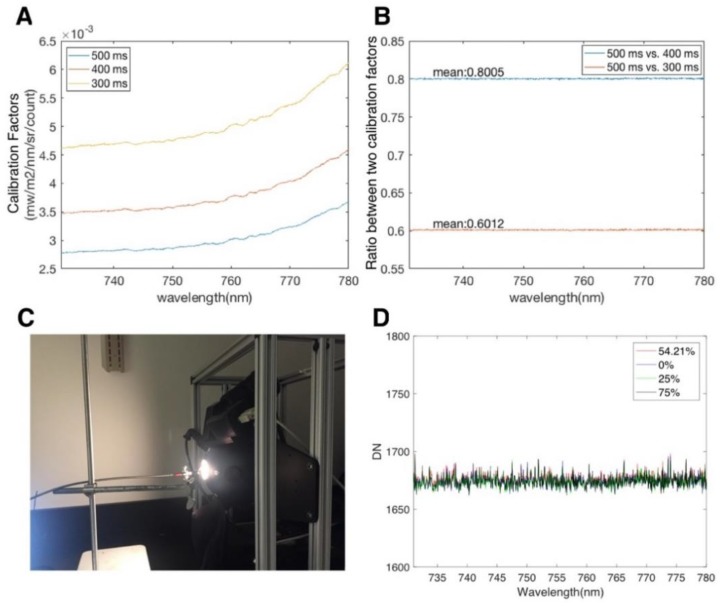
Lab tests of FluoSpec 2. (**A**) Calibration Factors estimated using FluoSpec 2 and the HELIOS integrating sphere. The calibration factors were collected under various integration times. (**B**) The ratio between calibration factors at 500 ms, 400 ms, and 300 ms. (**C**) Lab set-up when calibrating FluoSpec 2 and testing whether the shutter is “light-tight”. (**D**) Digital numbers (DNs) measured at various light intensities. The attenuator is fully open at 0%, and the light intensity in this setting is twice the intensity of peak solar radiation.

Either a laptop (Windows system) or a Raspberry Pi 3 (Linux system) can be used to control the system. FluoSpec Manager, a software that controls the system on a laptop, is written in Visual Basic with libraries provided by OceanOptics (OMNI SPAM, Figure 2). FluoSpec Manager allows the user to control the time interval of measurements and to set the integration time (automated or manual). When power consumption is an issue in some sites, the laptop is replaced with a Raspberry Pi 3 which is essentially a single-board computer running a Linux system (Raspbian). Codes written in C++ are run based on the open source driver, SeaBreeze, to control the system. Given that the Raspberry Pi 3 is much cheaper than a laptop, we recommend using Raspberry Pi in the future.

We use a simple algorithm to optimize the signal, which ideally needed to be near the middle of the dynamic range to avoid saturation. Given the high signal-to-noise ratio (SNR) of QEpro, one can assume that the raw signal received by the spectrometer (DN) is linearly correlated with the integration time. Thus, during the optimization, the program first collects a spectrum at the default integration time and calculates a scaler, which equals the maximum 100,000/DN. Then, the integration time of the actual measurement is calculated as the default integration time × the scaler. Data are acquired in the following sequence: for every 10 min, we first optimize the irradiance measurements and then use the optimized integration time to measure the irradiance. Radiance measurements are then optimized and measured in the same way.

The dark signal from the spectrometer is measured before the field installation under various temperature values and integration times. The assumption is that for a given TEC temperature, the dark current is linearly correlated with the integration time. Thus, it is possible a create look-up-table (LUT). The dark current is subtracted from each measurement, which can give us the real signal from the sky or vegetation.

### 2.3. Calibrations and SIF Retrieval

Before the system was used in the lab or field tests, a few calibration and modifications were needed to ensure the highest data quality was obtained. First, a wavelength calibration was needed to make sure that the spectra from both channels (solar and vegetation) were aligned. It is possible to do this with a light source with known emission peaks, for example, a mercury–argon light. Argon has at least four strong emission peaks in the 730 to 780 nm range to ensure proper wavelength calibration. Essentially, wavelength calibration establishes a polynomial relationship between the pixel number of the CCD detector in the spectrometer and the actual wavelength:

λ_p_ = λ_0_ + C_1_p + C_2_p^2^ + C_3_p^3^, (3)

λ_p_ is the wavelength of pixel number p. λ_0_ is the wavelength of the first pixel. C_1_, C_2_, and C_3_ are coefficients that can be derived from the calibration. During the calibration, λ_p_ and p are recorded, and the rest of the parameters are derived from the least square optimization. An excel spreadsheet for calculating the wavelength calibration parameters is available on Github (the link is available in the acknowledgement).

Radiometric calibration of FluoSpec 2 needs to be done on both the fiber connected to the upward cosine corrector and the downward bare fiber that points to the vegetation canopy. Calibration of the bare fiber is done with a homogenous light source with known intensity. We recommend using an integrating sphere, and in our case, the Labsphere HELIOS 8 inch integrating sphere (Labsphere, North Sutton, NH, USA) was used. The raw data and dark current at various integration time were recorded, and the calibration factor was calculated as

(4) $$Cal_{BF}=\frac{RAD}{Raw-Dark},$$

*RAD* is the radiance of the light source, which is provided by the integrating sphere. *Raw* is the digital number measured by FluoSpec 2 when the light source is on and the shutter of QEpro is off. *Dark* is the dark current measured when the internal shutter of QEpro is closed. *Cal_BF_* is the calibration factor, which is a function of wavelength and integration time. We made measurements under various integration times to examine whether the calibration factor obtained at one integration time can be used to calculate the calibration factor at other integration times. This is important because field measurements are made at various integration times (see Data Acquisition). Similarly, we calculated the calibration factor for the cosine corrector using a tungsten–halogen light source (HL-3P-CAL, OceanOptics, Inc.). Once the calibration is done, it is important to keep the light path intact, i.e., fiber optics should not be detached from the spectrometer and the shutter. Alternatively, FluoSpec 2 can be assembled in the field, and calibrated using the cross-calibration approach below.

**Figure 3:**
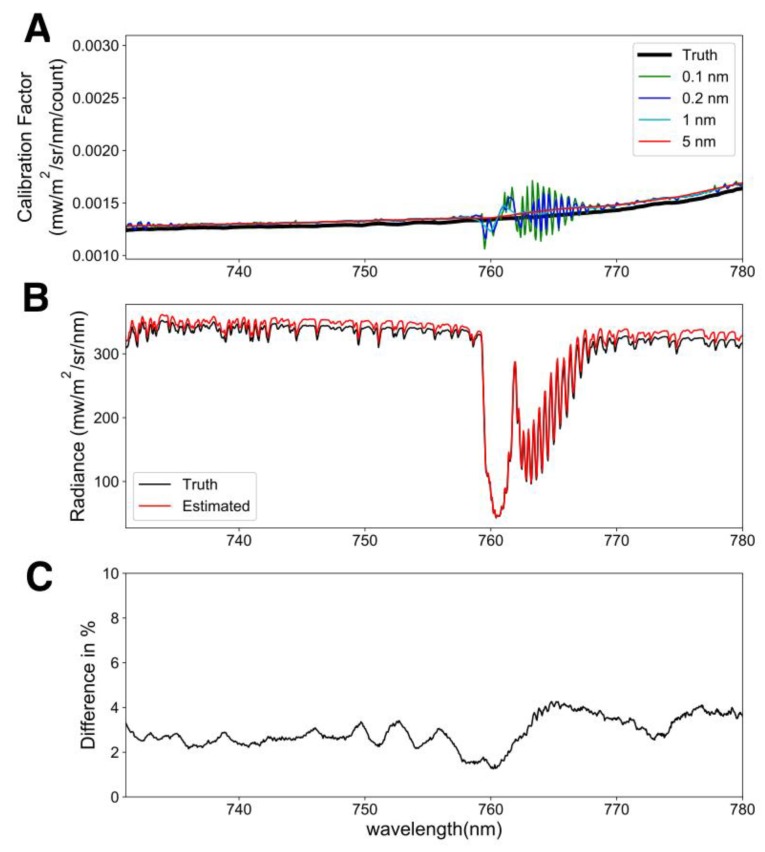
Field calibration of FluoSpec 2. (**A**) Calibration factors at four different spectral resolutions, compared with the true calibration factor obtained in the lab with an integrating sphere. (**B**) Comparison between the radiance spectrum estimated with lab calibration and the spectrum estimated with cross calibration. (**C**) Relative difference between the truth and the estimated spectrum.

In-field radiometric calibration of FluoSpec 2 can be challenging. An integrating sphere as large as HELIOS is difficult to deploy in the field. It is even more challenging once instruments are installed on tall towers with limited space on the top. Here we provide a relatively simple approach–a cross-calibration methodin which a separate spectrometer that has been calibrated with the integrating sphere in the lab is used as a reference to calibrate FluoSpec 2. In the field, both the calibrated spectrometer and FluoSpec 2 measure a spectralon panel at the same time. The calibration factor of FluoSpec 2 is then calculated as the ratio between the radiance measured by the reference spectrometer (Rad in Equation (4)) and the DN from FluoSpec 2 (Raw–Dark in Equation (4)). Since the spectral resolution of QEpro is high, there is an obvious spectral shift between spectrometers, which results in a wiggle-like artefact in the O_2_A band in the calibration factor spectrum (Figure 3A). The true calibration factor spectrum is a smooth curve with no obvious wiggles. To deal with this issue, a moving window was applied to the reference radiance and the DN from FluoSpec 2. The key assumption here is that even though the original radiance spectra (particularly near 765 nm) have obvious structures, the calibration factor curve actually is smooth. Thus, we can use the smoothed spectra from FluoSpec 2 and the reference spectrometer as inputs to Equation (4) to calculate the calibration factor. We estimated the uncertainty of this approach using two spectrometers which were both calibrated using HELIOS in the lab. We then measured a spectralon panel using one of them as a reference, and the other one as a spectrometer to be calibrated. We tested various sizes of the moving window (0.1 nm, 0.2 nm, 0.5 nm, 1 nm, 2 nm, 5 nm, and 10 nm) against the truth from the HELIOS calibration.

Another important note about the spectrometers is their nonlinearity. Nonlinearity refers to the issue that the response of the spectrometer to radiance is not linear across the entire dynamic range (in the case of QEpro, 0 to 200,000). The sensitivity of the detector, *α*, is defined as the change in DN (Δ*DN*) resulting from a change in signal at the detector, Δ*I*:

(5) $$\alpha =\frac{\Delta DN}{\Delta I},$$

For a perfectly linear system, *α* is constant, irrespective of the light intensity. However, most spectrometers are nonlinear to a certain degree, which results in varied levels of sensitivity at different light intensities. QEpro is close to linear when the DN is around 100,000. However, if a strong absorption feature is needed in SIF retrieval, such as the O_2_A band, the range of DN from the inside to the outside of the absorption can be from 10,000 to 100,000. Thus, correction for nonlinearity is necessary. The nonlinearity of this system was characterized by the manufacturer and a set of nonlinearity correction factors is included. It is recommended that the raw data is always corrected for nonlinearity:

*DN_cor_* = *D* + (*S* − *D*)/(*C*_0_ + *C*_1_(*S* − *D*) + *C*_2_(*S* − *D*)^2^ + … + *C*_7_(*S* − *D*)^7^), (6)

where D is the mean values of the dummy pixels, and S is the signal.

We used both the Spectral Fitting Method (SFM) and Singular Vector Decomposition (SVD) to retrieve SIF at 760 nm [46]. Given the high spectral resolution of this spectrometer, SIF can also be retrieved with only Fraunhofer lines with the SVD approach [12,44].

### 2.4. Lab and Field Tests

We assessed the dark current of the spectrometer under various integration times and detector temperatures. We used 10 ms, 100 ms, 200 ms, 500 ms, 1 s, 2 s, and 3 s as integration times and −10 °C, 0 °C, 10 °C, and 20 °C as detector temperatures.

We also tested whether the system was “light-tight” after QEpro had been installed in the TEC enclosure. Particularly, we assessed whether the internal shutter completely blocked the light and whether the FOS 2 × 2 TTL shutter leaked light when the shutter was completely closed. We tested this by pointing the fiber optic cable to the HELIOS light source, whose light intensity can be changed with a Variable Attenuator (VA). When the VA is fully open (0% closed), the light intensity is two times stronger than the sunlight at noon. We tested this with the VA closed at 0%, 25%, 54.21%, 75%, and 100%. We then assessed the extent of change in DN seen across the spectrum. We tested whether FluoSpec 2 could detect the well-known Kautsky Effect–the phenomenon in which fluorescence changes when a vegetated surface is exposed to incoming radiation after a long period (at least 30 min) in the dark. Fluorescence should first rapidly increase and then gradually decrease. We used a blue LED light, which emitted photons outside the range of detection of FluoSpec 2, as the light source. A potted plant (*Pachira aquatica*) was kept in the dark for 30 min. We started the measurements with the LED light off and then turned on the blue LED after 20 s. Data were collected every second.

## 3. Results

In this section, we first show the results from radiometric calibrations done in the lab and field, tests on the light leak of FluoSpec 2, and the relationships between dark signals and temperature and integration time. We then present an example of the SIF measurements in the field and a network of ground SIF observations (FluoNet) that has been established.

The results from the estimation of calibration factors under various integration times suggest that we can normalize calibration factors with the integration time. The calibration factors collected at different integration times (300 ms, 400 ms, and 500 ms) share the same shape (Figure 2A), and it is clear that the calibration factor is a function of integration time (Figure 2B). We show that the ratio between two calibration factors scales with the inverse of the integration time:

(7) $$\frac{Cal_{1}}{Cal_{2}}=\frac{IntTime_{2}}{IntTime_{1}},$$

For example, the ratio between calibration factors collected at 500 ms and 400 ms is ~0.8 (400/500). This means that we can normalize the calibration factor using the integration time, and use the calibration factor collected at one integration time to estimate the calibration factors at other integration times. Since the integration time is optimized and may change in every measurement cycle (10 min), the result here demonstrated that a single calibration factor normalized by the integration time can be used in Equation (4) to calculate Radiance = Cal × (Signal − Dark).

It is important to test the light leak of a spectroscopy system as leaks represent noise from other sources. We found that FluoSpec 2 is “light-tight” when the spectrometer is kept in the enclosure (Figure 2D). It is also important to use fiber optics with a metal or black jacket, which help to block ambient light. Potential components along the light path between the tip of the bare fiber and the detector at the spectrometer include the shutter and the spectrometer itself. When we closed the shutter and pointed the fiber to the port of an integrating sphere (Figure 2C), an incoming radiation two times the peak solar intensity caused a change in the raw DN of 3–4 across the spectrum (1 s integration time). That means if any leak had occurred, it was about 0.003% of the actual signal (in comparison, SIF was about 1~5% of the signal).

The in-field calibration uncertainty was less than 5%, on average (Figure 3). For the sake of simplicity, we only show the performance (difference in % radiance) from the 5 nm moving window. The assumption of a smooth calibration factor is valid as we can see from the true calibration factor in Figure 3A. The artefact in the calibration factor was obvious when the spectral resolution was high, especially in the O_2_A band. We hypothesize that this is related to (1) a small spectral shift between two spectrometers; (2) a lower SNR in the O_2_A band. When the moving window was 5 nm, the fine structure in the O_2_A band was gone, and the calibration factor was close to the true values. Comparing the true radiance with the estimated radiance from the in-field calibration shows that the error was on average 2.94% of the total radiance value and varied from 1.25% to 4.25%.

**Figure 4:**
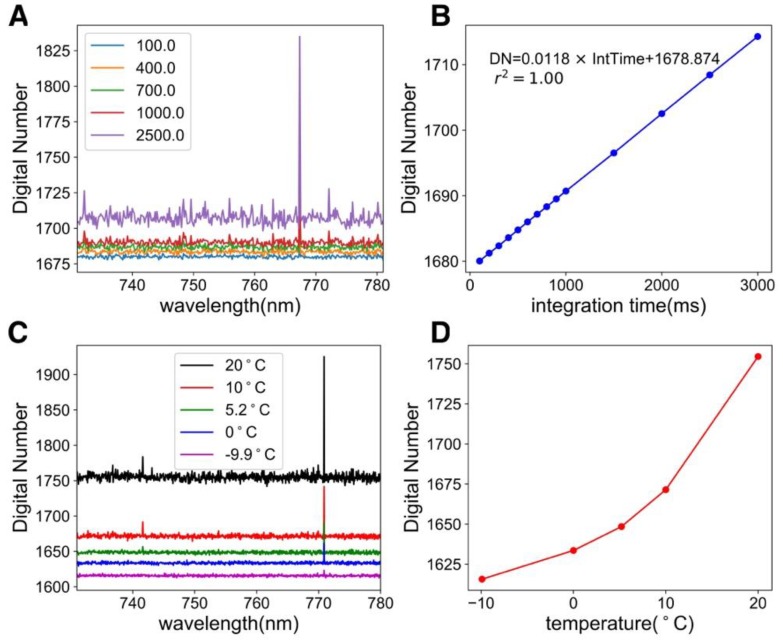
(**A**) Dark current under various integration times (100 ms, 400 ms, 700 ms, 1000 ms, and 2500 ms). (**B**) The relationship between integration time and the dark current. (**C**) Dark current collected at different TEC temperatures. (**D**) Non-linear relationship between temperature and dark current, indicating the importance of keeping a stable detector temperature.

We demonstrated that the dark current is a linear function of the integration time under a fixed temperature (Figure 4). In Figure 4A, we measured the dark signal under various integration times (100 ms, 400 ms, 700 ms, 1000 ms, and 2500 ms) at −10 °C. First, the variation of DN in each dark spectrum was small (~0.06%), suggesting a near uniform dark response. Second, it was possible to characterize the dark DN by the integration time. We also show the importance of keeping the detector temperature low and stable in Figure 4C,D. The difference between 20 °C and −10 °C in the dark current at an integration time of 100 ms was ~120. The changes in DN as temperature rose were nonlinear. This result implies that there is no need for routine dark measurements when the spectrometer is kept at a stable temperature (e.g., −10 °C). A look-up-table (LUT) that stores the dark current under various temperatures and integration times could be created before the field installation and used in the post-processing.

**Figure 5:**
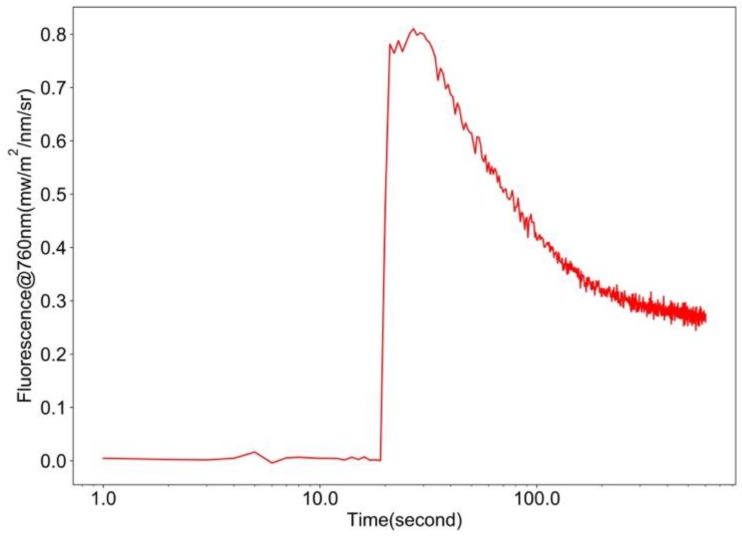
Kautsky Effect detected by FluoSpec 2 in the lab using a blue light-emitting diode (LED) light.

**Figure 6:**
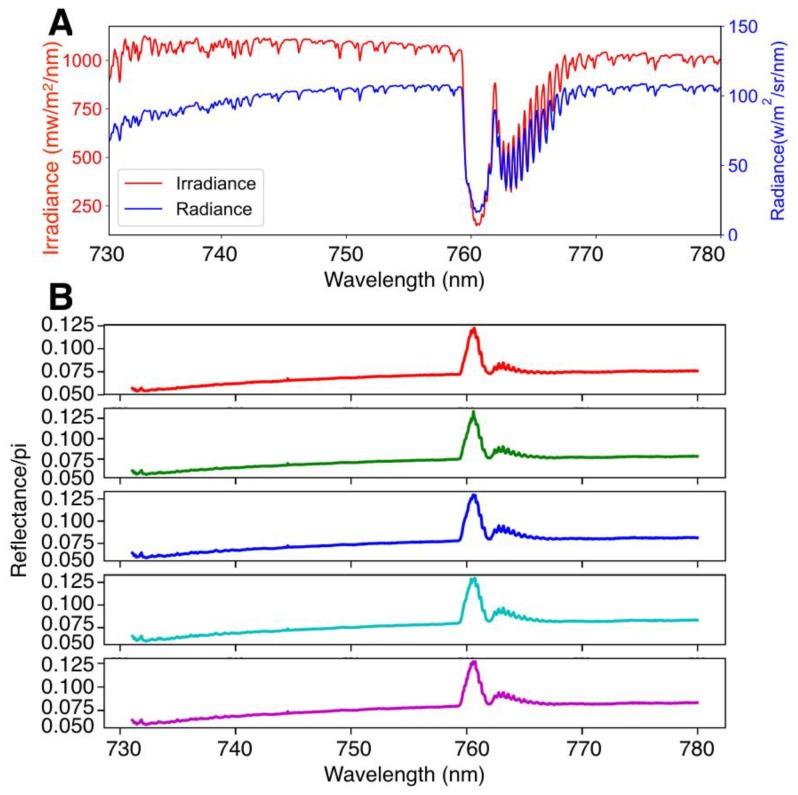
An example of data collected by FluoSpec 2 at noon. (**A**) Irradiance collected by the upward-looking cosine corrector and radiance collected by fiber optics pointing to the tree canopy. (**B**) Five consecutive reflectance spectra collected in ten minutes, calculated as radiance/irradiance.

We used the Kautsky effect as a test of the sensitivity of FluoSpec2 (Kaustky and Hirsch, 1931). A blue LED light was used as an excitation light source to induce fluorescence. A clear fluorescence peak and a gradual relaxation of fluorescence were detected by FluoSpec 2 in the lab (Figure 5). Examples of field irradiance, radiance, and reflectance data collected within ten minutes are shown in Figure 6.

**Figure 7:**
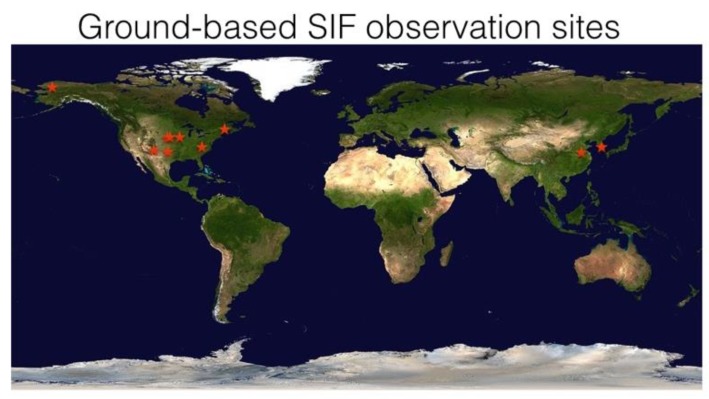
FluoNet. A network of sensors that measure SIF. The authors are also aware of installations conducted by other researchers in various types of ecosystems. Like FLUXNET, a global network of SIF measurements will benefit the scientific community.

**Figure 8:**
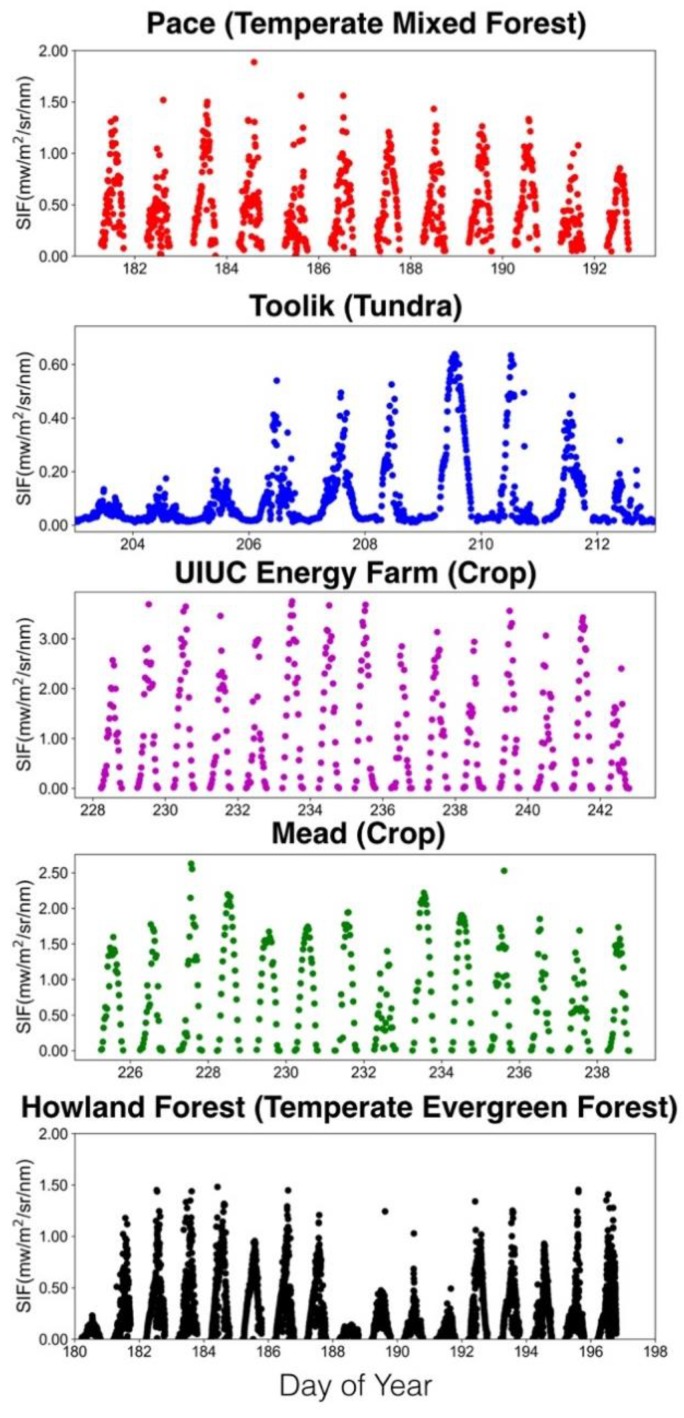
Examples of SIF collected during the growing season at four representative ecosystems.

A network of FluoSpec 2, FluoNet, has been established in various types of terrestrial ecosystems in the past a few years, including grassland, forests, and agricultural fields (Table 1 and Figure 7). Figure 8 shows SIF data collected at four representative ecosystems during a growing season. SIF data have a clear diurnal pattern, and also a day-to-day variation as a result of changes in incoming radiation (cloudiness). During the summer at the Alaska tundra site, we observed a low (~0.05 mw/m^2^/nm/sr) but non-zero fluorescence from 10p–5a as a result of the midnight sun. In Howland, Maine, a higher sampling rate (every minute) results in a denser time-series. Across the biomes, SIF is highest in crop and lowest in tundra (which has 24 h’ sunlight during the measurement period); temperate mixed forests and evergreen forests have similar magnitudes of SIF. The magnitudes of SIF values observed by FluoSpec 2 at various ecosystems were consistent with satellite-based retrievals [8,9,47]. This international ground remote sensing network is an important data sources for ecosystem research, combined with some regional in situ spectral networks under development, including EuroSpec [48] and ChinaSpec [49], and a few individual sites where SIF observations are being made or planned.

## 4. Discussion

Continuous and long-term observations of ecosystem functioning can provide important insights into ecosystem responses to environmental drivers and constraints to ecosystem models. Photosynthesis is one of the key ecosystem processes and its variations over space and time provide valuable information on the carbon, water, and nutrient cycles [50]. Automating measurements like SIF and canopy reflectance can also allow a shift in the focus on measurements which are equally important but difficult to measure automatically [51]. A network of these sensors is a tool to allow the understanding of inter-ecosystem differences in plant functioning, responses to environmental stresses, and up-scaling to regional and global scales.

We showed that, with careful design, continuous observations of SIF could be achieved in various types of ecosystems. The following are key issues to address when designing the system: (1) the use of a spectrometer with a high signal-to-noise ratio (SNR, ~1000:1 @755 nm). It should be noted that the SNR during actual measurements varies from wavelength to wavelength and is also related to the changes in the signal, i.e., solar radiation. It also needs a well-documented nonlinear, spectral resolution, fine enough to resolve Fraunhofer lines. QEpro represents a significant advancement compared with other OceanOptics spectrometers, including HR2000+. (2) stable temperature control is required. We showed that with the temperature control, background noise drops dramatically (Figure 4C). Considering that the SIF at 760 nm is only 1~5% of the incoming radiation at 760 nm, a small improvement in noise reduction can be significant for SIF retrieval. (3) Humidity control is required. Under high humidity (relative humidity >80%), we found that there were significant changes in the spectra, shown as wiggles randomly appearing along the spectra as a result of water condensation on the detector (data not shown). The spectrometer needs to be better protected from humidity than many other field instruments (e.g., dataloggers) as it is not designed for outdoor use.

This automated system has been evolving and improving since its first measurement in 2012 at Harvard Forest [2]. Steps have been taken to make the system more reliable in complicated environments. Additional changes will be made in the near future. For example, a multi-angle system would allow observations of more individuals and an estimation of the angular effect of SIF. As a remote sensing measurement, it is subjected to sun-sensor-object geometry [52]. Measurements are subjected to both leaf physiological controls [53,54] and structural effects [55,56]. It is important to disentangle these two effects on the temporal variations of SIF to answer the question whether whether the changes are a result of changes in the fraction of sunlit and shaded leaves, or leaf-level physiology, such as varying non-photochemical quenching [12,54]. Measurements of multi-angle SIF and canopy reflectance may allow us to solve this issue using existing radiative transfer theories [52,55,57].

Several important research questions can potentially be addressed with FluoNet: (1) the diurnal variations of SIF, photosynthesis, and non-photochemical quenching (NPQ). As the three main pathways for absorbed photosynthetically active radiation, the variation in SIF, photosynthesis, and NPQ provide information on the stress-related changes in electron transport. Combining SIF, EC, and PRI measurements may help identify how the plant canopy as a whole uses absorbed photons. (2) GPP-SIF relationship across different biomes. FluoNet can help understand whether there is a biome-dependent relationship between SIF and GPP [58] or a universal GPP-SIF relationship globally [17]. More importantly, it can help us to understand the mechanism that drives this relationship, whether vegetation structure is the dominant control, what the time scale is, and whether the changes in the light use efficiency and yield of fluorescence (SIF_yield_) play important roles. (3) FluoNet can help us to understand the structural and biological controls of SIF signals. FluoNet aims to be inclusive and involve the efforts from groups that have designed other types of SIF systems. The overall goal is to enable an effective network to share ideas and data with the entire scientific community.

A few existing and future satellite programs can be used to retrieve SIF, including GOSAT, GOME-2, OCO-2, OCO-3, TROPOMI, FLEX, and GeoCARB [8,59,60,61]. Most of the satellites take a snapshot at a certain time of day (e.g., OCO-2 local time is around 1:30 p.m.), except GeoCARB, which is a geostationary satellite that can provide multiple observations in a day, and OCO-3, which will be installed on the International Space Station. Ground observations of SIF by FluoSpec 2 are thus instrumental to evaluate satellite SIF products [2]. FluoSpec 2 and similar sensors thus have their own niche by providing measurements at a high temporal frequency, which provide tools to study photosynthesis-related responses to plant stress [10,62,63,64,65]. FLUXNET, a global network of micrometeorological flux towers has opened a new era for ecosystem studies and has significantly contributed to scientific advancement in terrestrial ecology. We envision that a network of FluoSpec 2 and similar sensors could further move the ecosystem studies to the next stage.

## Figures and Tables

**Table 1:** SIF Network (FluoNET): site names, coordinates, and plant functional types (PFTs). * Kessler Atmospheric and Ecological Field Station ** Jurong Agricultural Ecosystem Station.

Site Name	Location	Geolocation	Plant Functional Type
Pace	Virginia, USA	37.9229, −78.2739	Temperate mixed forest
Toolik	Alaska, USA	40.6223, −76.1222	Tundra
MPJ	New Mexico, USA	34.4385, −106.2377	Pinon-Juniper
UIUC Energy Farm	Illinois, USA	40.0658, −88.2084	Crop (soybean)
Mead 1	Nebraska, USA	41.1649, −96.4701	Crop (maize/soybean)
Mead 2	Nebraska, USA	41.1797, −96.4397	Crop (maize/soybean)
Howland	Maine, USA	45.2041, −68.7402	Temperate evergreen forest
KAEFS *	Oklahoma, USA	34.9846, −97.5223	Grass (prairie; C3/C4)
Cheorwon	Gangwon, Korea	38.2013, 127.2506	Crop (paddy rice)
CN-JRO **	Jiangsu, China	31.8068, 119.2173	Crop (paddy rice)
CN-SHQ	Henan, China	34.5203, 115.5894	Crop (Wheat/Maize)
